# Alarming Dental Hæmorrhage

**Published:** 1869-12

**Authors:** N. H. Church

**Affiliations:** Patoka, Ind.


ARTICLE IV.—*Alarming Dental Hæmorrhage.* By N. H. CHURCH, M.D., Patoka, Ind.

UNDER the above caption Dr. A. I. Edison, Colchester, Illinois (*Am. Jour. Dent. Sciences*, Aug., 1869), details a case of alarming hæmorrhage from the extraction of middle and posterior molars of Inf. Max., right side. Hæmorrhage lasted in the neighborhood of twenty-four hours before it was arrested.

Sunday, September 26, was called upon to visit Mrs. Mc——, who was suffering from odontalgia of bicuspid tooth of Sup. Max., left side. I extracted tooth. Hæmorrhage, at the time, was no more than usual.

October 2, P.M., was called upon by Mrs. Mc——, who was "alarmed about the bleeding tooth." I ascertained that hæmorrhage had been going on, unabated, since the extraction of the tooth. I placed a powder of tannic acid and gum catechu—equal parts—in the bleeding cavity. Kept *in situ* with compress of cotton saturated in muriatic tincture of iron.

Patient went home, and I heard no more from her until Sunday, October 3, A.M., when I was summoned to put in an appearance at her residence at once, and, if possible, arrest the hæmorrhage, as it was quite profuse. I carried with me a small phial of Monsel's solution of the per-sulphate of iron.

Arrived at the house, found patient very much exhausted. Learned that hæmorrhage had been greater during the night previous than it had through the whole week past. After having applied a few compresses of cotton saturated in the Monsel's solution—the last one being held *in situ* with the finger for the space of half an hour—the hæmorrhage had entirely ceased.

I dare not express myself as to the amount of blood lost in this case, lest the readers of this article think me rather too hæmorrhagic.

The patient came up to her usual standard of health, in a few days, without after-treatment.

